# Estimating Genetic Variability in Non-Model Taxa: A General Procedure for Discriminating Sequence Errors from Actual Variation

**DOI:** 10.1371/journal.pone.0015204

**Published:** 2010-12-06

**Authors:** Karen Dawson, Roger S. Thorpe, Anita Malhotra

**Affiliations:** School of Biological Sciences, College of Natural Sciences, Bangor University, Bangor, Gwynedd, United Kingdom; Aarhus University, Denmark

## Abstract

Genetic variation is the driving force of evolution and as such is of central interest for biologists. However, inadequate discrimination of errors from true genetic variation could lead to incorrect estimates of gene copy number, population genetic parameters, phylogenetic relationships and the deposition of gene and protein sequences in databases that are not actually present in any organism. Misincorporation errors in multi-template PCR cloning methods, still commonly used for obtaining novel gene sequences in non-model species, are difficult to detect, as no previous information may be available about the number of expected copies of genes belonging to multi-gene families. However, studies employing these techniques rarely describe in any great detail how errors arising in the amplification process were detected and accounted for. Here, we estimated the rate of base misincorporation of a widely-used PCR-cloning method, using a single copy mitochondrial gene from a single individual to minimise variation in the template DNA, as 1.62×10^−3^ errors per site, or 9.26×10^−5^ per site per duplication. The distribution of errors among sequences closely matched that predicted by a binomial distribution function. The empirically estimated error rate was applied to data, obtained using the same methods, from the Phospholipase A_2_ toxin family from the pitviper *Ovophis monticola*. The distribution of differences detected closely matched the expected distribution of errors and we conclude that, when undertaking gene discovery or assessment of genetic diversity using this error-prone method, it will be informative to empirically determine the rate of base misincorporation.

## Introduction

The study of naturally occurring genetic variation, whether between species [1], [2], populations [3] or individuals [4], is of vital importance in biology. The rich natural diversity of peptides [5]–[7] and other natural products, of considerable interest for drug development and other biotechnological applications, can result from isoforms coded by the same genetic locus or the duplication of genes to form related multi-gene families. Although new generation sequencing technology has the power to make rapid advances in the discovery of genetic variation underlying this diversity, for many non-model taxa, more traditional approaches which combine PCR using conserved regions of a known gene with cloning to separate the mixed product (resulting from amplification of heterozygous loci or simultaneous amplification from multiple loci) are still widely used [8], [9]. Although the finite error rate of *Taq* and other polymerases has been known for some time [10]–[12], in most applications it is not significant. However, sequences resulting from cloning of mixed PCR products will be particularly susceptible to PCR error because cloning isolates a single copy of the target sequence after many cycles of PCR; any mistakes present in this copy will be perpetuated in all subsequent steps. In addition, mixed-template PCR products are known to have higher error rates due to interactions between templates [13]. Multigene families frequently evolve rapidly through birth and death processes [14], [15], [3] and in non-model species without sequenced genomes, the exact number of different gene copies carried by a particular individual may not be known in advance. It is therefore important that the rate of misincorporation be quantified, and appropriate statistical analysis employed to help discriminate between genuine evolved sequences and PCR artefacts. Inadequate discrimination could lead to incorrect estimates of gene copy number, population genetic parameters, phylogenetic relationships and the occurrence of gene and protein sequences in databases that are not actually present in any organism. However, studies employing these techniques rarely describe explicitly how errors arising in the amplification process were detected and accounted for.

Estimates for the error rate of *Taq* polymerase available in the literature [11], [12], [16] vary somewhat and will be affected by PCR conditions, especially cycle number [17] and the form of polymerase used. Rather than using an estimate based on such variable numbers, here we determine the method-specific error rate for the conditions and enzyme preparations used in our ongoing studies of gene discovery in the Phospholipase A_2_ (PLA_2_) toxin family of pitvipers, by applying them to a single copy homoplasmic gene [18], mitochondrial cytochrome *b* (MT-CYB) from the mountain pitviper *Ovophis monticola*. Using this empirically determined error rate, we compared observed sequence differences in the toxin genes to the expected distribution of errors generated by a binomial probability mass function [19].

## Results

We obtained partial MT-CYB sequence (253 to 761 base pairs, averaging 715 bp, total 58592 bases) from 82 clones of PCR product amplified from a single individual (consensus sequence is available on GenBank, accession number HQ325161). Errors were defined as base pair inconsistencies between an individual sequence and the most common base pair found in that position in the alignment of all sequences. A total of 95 errors were observed, yielding a total error rate of 1.62×10^−3^ errors per site (0.162%) for the reaction conditions used here. The majority (96.8%) were substitutions, with a small number of single nucleotide deletions (3.2%). 73% of all sequences had one or more errors, 87% of which were transitions, with the substitution AT to CG comprising 71.7% of these. This is consistent with patterns seen in other studies [11], [12] and suggests that observed sequence differences arise from base misincorporation by *Taq* rather than from mtDNA heteroplasmy or amplification of NUMTs [20]. These figures were converted into per-duplication misincorporation rate (*m*) according to the formula of Hayes [21], *m = 2(f/d)*, where *f* is the frequency of errors observed in the PCR product and *d* is the number of doublings [12]. Only the pre-cloning cycles are relevant to calculating error rates as the second PCR is based on a large number of copies of the target sequence from the bacterial colony, and errors occurring after this point are unlikely to be seen in the final sequence data. Thus, we estimate *m* as 9.26×10^−5^ per site per duplication, based on the assumption of a doubling in every cycle. However, Kobyashi *et al.* [11], who reported a similar rate of 7.3×10^−5^ per site per duplication, quantified the amount of DNA present before and after PCR and found that only 16.6 doublings occurred in 25 cycles of PCR, since in later cycles the number of copies tends to plateau. Thus, our estimates are likely to be conservative.

Cummings *et al.* [19] suggested the use of a binomial probability mass function to calculate the probability of a sequence of given length possessing a specific number of errors under conditions with a known base misincorporation rate. Using the average sequence length of 715 bp and the method-specific error rate obtained from this experiment, the observed distribution of errors is close to the expected distribution generated by the binomial function (χ^2^ test, *P* = 0.765).

The same experimental methods were then used to amplify and sequence genes from the multigene venom PLA_2_ genes from a further two specimens of *O. monticola*, yielding 79 sequenced clones for B664 and 67 for ROM 39382. The resultant sequences were placed into groups based on sequence similarity using UPGMA and a consensus sequence generated based on the most common base at each position within each group. In both specimens, the sequences could be grouped into two main classes based on number of base pair differences (>100 bp difference between groups, <10 within each group). In B664, 10 samples were more distinct from either main group. Of these, 7 contained conserved features found in both groups in various positions and are likely chimeric duplication artefacts (and are not discussed further here), while 3 appeared to be highly distinct sequences with no clear relationship with any of the groups and are likely to represent separate alleles. Consensus sequences are deposited in GenBank (accession numbers HQ389258-61). The number of nucleotide differences (SNPs and indels) from the within-group consensus was counted, as direct pairwise comparison of error-prone sequences will inflate estimated divergence. Within each group, a sliding window analysis of the average number of substitutions per site, performed using DNAsp 4.10.9 [22], did not show any clear biases. This is consistent with random misincorporation errors as variability between sequences present in the genome is expected to be higher in regions known to undergo rapid evolution, such as the exons of venom coding sequences [6]. The expected frequency distribution of sequences showing specific numbers of errors was calculated using the binomial distribution. With 82 similar sequences under consideration, a sequence of this length would need to show 6 differences before it would be considered likely to be an actual polymorphism. A χ^2^ test showed no significant difference between the expected distribution of errors and the observed pattern of deviations (Table 1).

**Table 1:** Deviations from the consensus sequence in the fully sequenced test samples for Phospholipase A_2_ genes from *Ovophis monticola*.

Sample code (no. of sequences)	Seq. length	Number of samples showing given no. of base differences from group consensus sequence: observed (expected)								X^2^ value	*P*
		0	1	2	3	4	5	6	7		
B664* group A (42)	1754	4 (2)	5 (7)	11 (10)	5 (8)	7 (7)	8 (4)	2 (2)	0 (1)	8.78	0.19
B664* group B (25)	1724	1 (2)	6 (4)	9 (6)	6 (6)	2 (4)	1 (2)	0 (1)	0 (0)	5.33	0.38
ROM 39382 group A (44)	1847	3 (2)	7 (7)	7 (10)	11 (10)	11 (7)	2 (4)	3 (2)	0 (1)	4.65	0.59
ROM 39382 group B (23)	1694	1 (1)	2 (4)	3 (6)	8 (5)	5 (3)	3 (2)	1 (1)	0 (0)	5.82	0.44

*a further 10 samples from B664 did not fall into either group or show sufficient similarity to one another to form a third group.

The expected frequencies are calculated based on the error rates calculated for MT-CYB (see text), under a binomial probability distribution using the average length of the sequence in that group. χ^2^ values for test groups are derived from a comparison of the number of sequences showing given numbers of deviations from the consensus sequence with the expected distribution of errors in a set of sequences of the same length.

## Discussion

We have demonstrated that the distribution of nucleotide differences obtained in the application of the PCR cloning method to multigene families is highly consistent with the expected distribution of sequencing errors estimated using the binomial distribution. Observed differences were not significantly different from expected, suggesting that this may be a generally-applicable method to reduce the false discovery rate due to errors in gene discovery programs using this PCR-cloning method. This method can be used to determine a “threshold” number of differences above which a sequence should be treated as a unique allele. Error rates can vary greatly between studies. For example, Cummings *et al.* [19] also used a proofreading polymerase and reported a lower error rate than in this study (1.85×10^−5^). The precise causes of our higher observed misincorporation rate cannot be determined, as several factors vary between studies, including the form of *Taq* enzyme used, concentrations of other components in the PCR, the temperature regime, the strain of cloning vector and the primers and target sequence. However, this variation serves to highlight the dangers of extrapolating expected error rates from previous work, underlining the need to obtain procedure-specific error rates, at least until such time as the causes of variability in error rates are better understood. The decision of how many substitutions must be observed to accept a sequence as representing genuine diversity rather than PCR error will, however, depend on not only the error rate and length of the sequence under consideration, but also on the number of clones screened and the number of genuine alleles present. In the case of single-copy genes, this problem may reduce to finding the single most probable sequence. However, in large multi-gene families in which the protein products are subject to strong diversifying selection, there may be quite a few distinct alleles present. For example, at least 10 copies of the venom phospholipase A_2_ genes with nucleotide similarities between 67-89% have been detected in *Protobothrops flavoviridis* [23], which is relatively closely related to *Ovophis*. The higher the number of genuine alleles present, the more chance there may be of rejecting a genuine allele as erroneous. This is not a problem that is restricted to this methodology; even in newer 454 pyrosequencing approaches, the rarer alleles will be more affected by sequencing errors (which are actually higher than in traditional Sanger sequencing) both as a consequence of receiving lower coverage and an increased probability of occupying a multi-templated bead with dissimilar templates [24].

Cummings *et al.* [19] suggest using the Bonferroni correction to constrain the occurrence of Type II errors. Here we propose instead the use of a modification of this test, a sequential Bonferroni correction [25]. The sequential and standard tests are equally capable of detecting a single significant case, but the sequential form is less likely to reject additional significant cases [25]. However, it is also important to consider that in many cases, pairs of recently diverged or highly constrained alleles may differ by only a few base pairs, far lower than the threshold for acceptance as genuine alleles under the criteria of the Bonferroni correction, leading to existing diversity being ignored. Exactly how to balance the relative risks of accepting erroneous alleles and rejecting genuine alleles is a decision for individual researchers, to be made based on the purpose of their study. It must also be stressed that even sequences accepted as being generated from real alleles are likely to include PCR errors, especially in long sequences (using the estimated error rate, an amplicon of 2000 bp is likely to be completely error free in less than 5% of cases), so acceptance as a potentially genuine allele does not indicate that the true base pair order can be deduced from a single copy. Additional analysis such as comparisons of sequences from multiple individuals as well as confirmation from additional PCR of the same template DNA [19] are still essential if the target sequence must be known with great accuracy, for example if it is to be used in the deduction of amino acid sequences.

## Materials and Methods

Whole genomic DNA was extracted from *O. monticola* liver tissue in 80% ethanol (ROM 39382 for MT-CYB and ROM 39382 and a specimen obtained from the trade, designated B664 for the PLA_2_ study) using the Sigma GenElute mammalian genomic DNA miniprep kit. A fragment of the MT-CYB gene was amplified using the primers Gludgmod2 [26] and H16064mod (5′-GGTTTACAAGAACAAYGCT-3′), modified from H16064 [27]. Initial PCR was performed using Accutaq LA DNA polymerase (Sigma Aldrich) and reaction conditions were 35 cycles of 94°C (30 s), 60°C (1 min) and 72°C (2 m), with an initial denaturation phase at 94°C for 3 min and a final extension phase of 72°C for 5 minutes. PLA_2_ genes were amplified using primers New PLA_2_5′ and New PLA_2_3′ (Table 2) with reaction conditions consisting of an initial denaturation stage of 2 min 30 s at 94°C, followed by 35 cycles of denaturation for 1 min at 94°C, annealing at 53.6°C for 1 min, 3 min extension at 72°C, and a single final extension of 20 min at 72°C. PCR product was cleaned using WIZARD ® DNA cleaning kits (Promega), an adenylation reaction performed and cloned using TOPO TA cloning kits ® (Invitrogen). Colonies were matured on LB Amp plates at 37°C and positive (white) colonies were transferred to wells of liquid LB Amp growth medium and incubated overnight at 37°C. Subsamples from each well were then used in a second round of PCR using Abgene PCR master mix with a non-proofreading polymerase (reaction conditions were 35 cycles of 94°C for 1 min, 53.6°C for 1 min and 72°C for 3 min, with an initial denaturation phase at 95°C for 15 min and a final extension phase of 72°C for 20 minutes). Colony (specific) PCR products were cleaned using Exonuclease I and shrimp alkaline phosphatase (SAP) [28] for the MT-CYB study. PLA_2_ products were first quantified against 100 bp ladder on 1% agarose gel, with the PLA_2_ gene corresponding to approximately 1800–2200 bp. Cleaned products sequenced by Macrogen inc. (www.macrogen.com). Due to the relatively large size of the PLA_2_ gene, it was necessary to sequence using multiple primers, and reconstruct the full sequence using overlapping regions.

**Table 2:** PCR and sequencing primers for pitviper Phospholipase A_2_ toxin genes.

Name	Direction	Location (approx)	Sequence (5′-3′)
New PLA5′	Forward	1–19	GTATTCATGCGGCCGCGGATCACCTGCCAGGAGGA*
PLA_2_ Up1	Forward	Upstream of position 1	TCGCTTGGAGAYGGGAAG*
iF	Forward	575–594	CATCTGCCATTAACCCTACAG
R for 5′	Reverse	710–729	GCCGCAGWAGCATCCGTAA
F for 3′	Forward	1402–1423	TTTTCCAATCWTGGGGTGCCAG
iR	Reverse	1650–1668	GGTTCTGGCTCGGGGAGC
New PLA3′	Reverse	2102–2129	GTAGATCTCTGGCACCTGTTTATTACTC*

Samples used various combinations of the following primers to obtain full length sequence. Asterisks indicate primers used for PCR. PLA_2_ Primers were developed in previous PLA_2_ sequencing work (Anita Malhotra, unpublished data) apart from Up1, which is a novel primer designed as an alternative to New PLA5′ in some amplifications. Sequence locations are derived from the aligned gene sequence data used in the analyses.
